# Serotonergic System Dysregulation in Alzheimer’s Disease

**DOI:** 10.1021/acschemneuro.6c00236

**Published:** 2026-07-15

**Authors:** Nour F. Al-Ghraiybah, Amer E. Alkhalifa, Dylan Spivey, Thomas Averill, Blake Engelkemier, Patricia Haro Lopez, Mary Grace Stuckey, Luke Jenkins, Amal Kaddoumi

**Affiliations:** † Department of Pharmacology and Toxicology, Medical College of Georgia, 160343 Augusta University, Augusta, Georgia 30912, United States; ‡ Department of Drug Discovery and Development, Harrison College of Pharmacy, 1383 Auburn University, Auburn, Alabama 36849, United States

**Keywords:** neurodegenerative disorders, Alzheimer’s disease, serotonin, serotonergic system, blood–brain barrier, gut-brain axis, neurotransmitters

## Abstract

Alzheimer’s disease (AD) is a progressive neurodegenerative disorder characterized by several key hallmarks, including the accumulation of amyloid-β (Aβ), neurofibrillary tangles (NFTs), neuroinflammation, and blood–brain barrier (BBB) dysfunction. Similar to other neurodegenerative diseases, AD involves disturbances in neurotransmitter homeostasis. However, the serotonergic system is complex, involving numerous mechanisms and pathways, which complicates the establishment of direct correlations with AD. This review examines the serotonergic system, focusing on alterations in serotonin (5-HT), its transporter, and its receptors in the context of AD, to identify current knowledge gaps and highlight ongoing research directions. It also emphasizes the role of 5-HT in vascular regulation and BBB integrity, and links the gut-brain axis to the serotonergic system.

## Graphical abstract

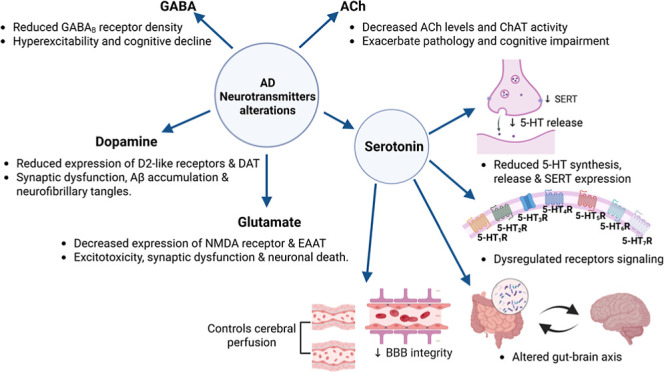

## Introduction

Alzheimer’s disease (AD) is a progressive neurodegenerative disorder that significantly affects the aging population.^1^ As the leading cause of dementia, AD accounts for 60–80% of all dementia cases worldwide.^2,3^ Globally, over 55 million people are estimated to be living with dementia, a figure projected to nearly triple by 2050 due to demographic aging and a lack of curative therapies.^4^ Clinically, AD begins with subtle symptoms such as episodic memory loss and impaired spatial navigation, progressing insidiously to involve deficits in executive function, language, behavior, and eventually, total independence.^5,6^ In its later stages, patients may experience severe neuropsychiatric symptoms, such as hallucinations, delusions, depression, and sleep problems, which further exacerbate caregiver burden.^7^

Pathologically, AD is characterized by the extracellular accumulation of amyloid-β (Aβ) peptides forming diffuse and neuritic plaques, and the intracellular aggregation of neurofibrillary tangles (NFTs) composed of hyperphosphorylated tau proteins.^8–10^ These lesions are predominantly distributed in the hippocampus, entorhinal cortex, and neocortical association areas, regions essential for memory and cognition, and are associated with synaptic loss, neuronal atrophy, and widespread neurodegeneration.^11,12^ In addition to these hallmark features, AD pathogenesis encompasses a spectrum of molecular abnormalities, including chronic neuroinflammation, oxidative stress, mitochondrial dysfunction, and blood–brain barrier (BBB) breakdown, all of which synergistically contribute to disease progression (Figure 1).^13–16^ While the neuropathological features of AD are well characterized, the mechanisms underlying disease initiation and progression remain under investigation.

**Figure 1:**
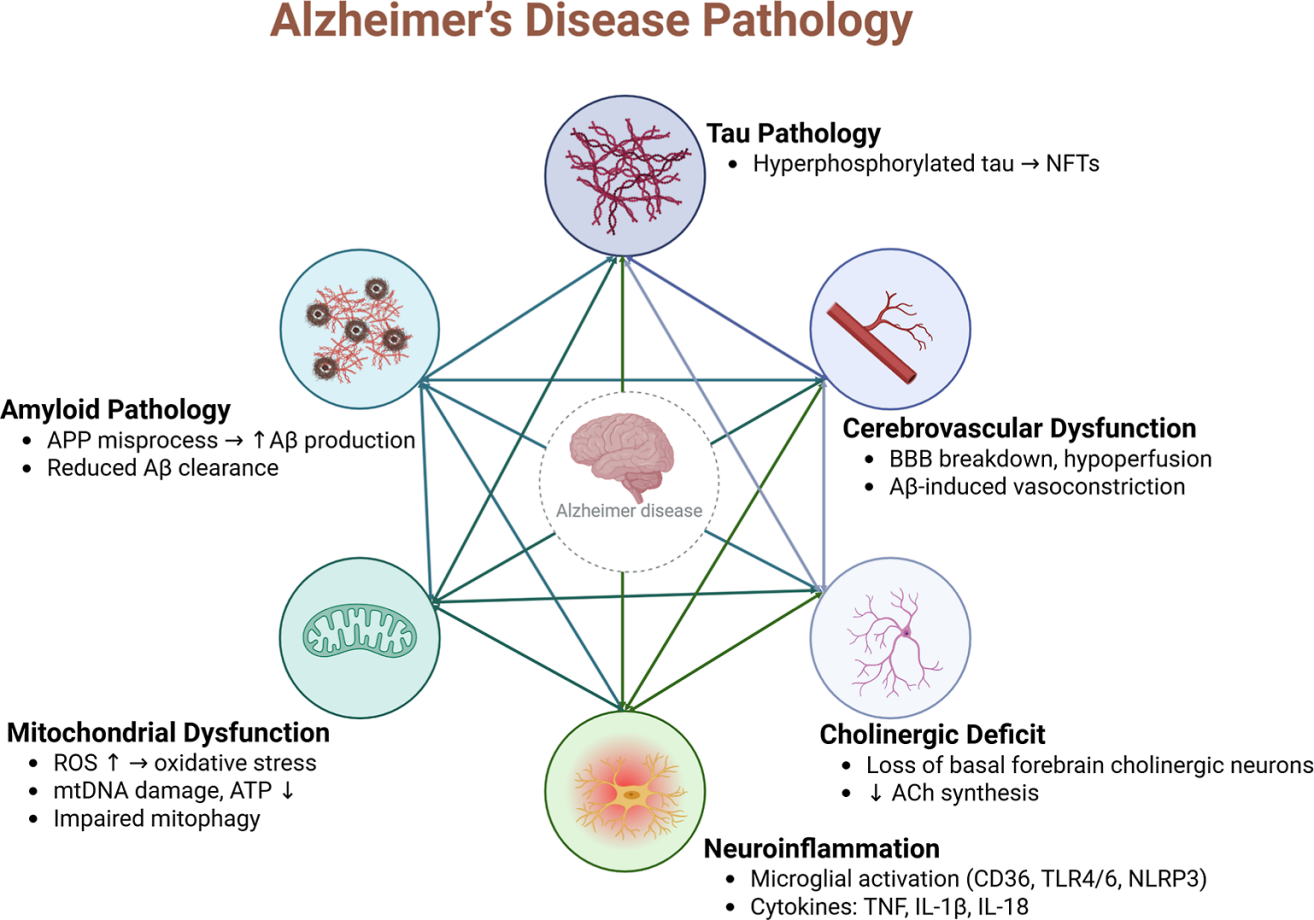
Overview of AD pathology. AD is characterized by multiple interrelated pathological hallmarks, including amyloid plaque accumulation, tau hyperphosphorylation, cerebrovascular impairment, cholinergic neuron loss, neuroinflammation, and mitochondrial dysfunction.

In AD, the neurotransmitter systems are also dysregulated and contribute to cognitive decline and neuropsychiatric symptoms. The cholinergic system is a primary site of dysfunction in AD pathology.^17,18^ Acetylcholinesterase inhibitors (AChEIs) can alleviate cognitive impairment in AD by preventing the breakdown of synaptic ACh.^19–22^ Agents such as donepezil, rivastigmine, and galantamine offer improved tolerability, greater central selectivity, and modest cognitive benefits in patients with mild-to-moderate AD.^23,24^ Other neurotransmitters, including dopamine, glutamate, and GABA, are also altered in normal aging and AD.^25–28^ In AD, the serotonergic system is also dysregulated and contributes to cognitive decline and neuropsychiatric symptoms.^29^ In the sections below, we review serotonin dysregulation in AD, including changes in serotonin function. This is followed by a discussion of serotonin receptors and transporters, with particular focus on receptor subtype-specific alterations in aging and AD. We also analyze the effect of serotonin on the BBB. Finally, we explore the interaction between serotonin and the gut–brain axis, emphasizing how peripheral serotonin signaling and interactions with the gut microbiota may impact central nervous system function and contribute to AD pathophysiology.

## Serotonin Dysregulation in AD

### Serotonin as a Neurotransmitter

Serotonin or 5-hydroxytryptamine (5-HT) is a monoamine that is produced from tryptophan, an essential amino acid. 5-HT is a neurotransmitter linked to cognitive, motor, and autonomic functions.^30,31^ 5-HT influences various physiological processes that affect mood, emotions, well-being, happiness, appetite, and sleep patterns.^29^ Furthermore, 5-HT functions as a mediator of motility within the gastrointestinal (GI) tract, where its release and serotonergic signaling via various receptors influence gastric emptying time, GI motility, secretion, and pathological alterations, as well as nausea and other GI hypersensitivities.^32,33^ Furthermore, 5-HT plays a role in tumorigenesis across various cancers, including gliomas, carcinoids, and carcinomas. 5-HT stimulates cancer cell proliferation and can promote angiogenesis by acting as both a growth stimulant and an angiokine.^34^

5-HT is synthesized in two steps; first, tryptophan is oxidized to 5-hydroxytryptophan (5-HTP) by the enzyme tryptophan hydroxylase 2 (TPH-2), then 5-HTP is converted into 5-HT by aromatic l-amino acid decarboxylase.^35–37^ The brain and the peripheral sites synthesize 5-HT separately. Two forms of tryptophan hydroxylase facilitate the synthesis of 5-HT: TPH-1, which is usually found in the pineal body and the digestive tract, and TPH-2, which is found selectively in the brain.^36^ In the periphery, 5-HT synthesis occurs in enterochromaffin cells in the mucosa of the GI tract and, to a lesser extent, in blood platelets.^38^ Following synthesis, 5-HT is released into the GI tract to facilitate digestive muscle contraction and peristalsis, or from platelets upon activation to aid neutrophil recruitment.^39,40^ In the CNS, 5-HT is synthesized and stored in presynaptic neurons in vesicles.^34^

In AD, there is a progressive degeneration of monoaminergic neurons, including noradrenergic neurons in the locus coeruleus and serotonergic neurons in the raphe.^41–43^ Human studies have revealed lower platelet 5-HT in patients with AD compared to those with subjective cognitive impairment. Additionally, reduced platelet 5-HT was associated with higher AD biomarkers in cerebrospinal fluid (CSF), specifically total tau and tau/Aβ_42_ ratio.^44^ Post-mortem brain tissue staining of the dorsal and median raphe nuclei in patients with AD showed a significant decrease in 5-HT neuron density: 41% in the dorsal raphe and 29% in the median raphe, compared to age- and sex-matched controls. However, no links were found between the reduction in 5-HT neuronal density and behavioral changes, suggesting that although the raphe nuclei are notably affected in AD pathology, the plasticity and complexity of the serotonergic system may partly explain the lack of a direct correlation.^45^

In mouse models of AD, similar serotonergic degenerations are observed. In APPswe/PS1Δ*E*9 mice, an AD mouse model with Aβ pathology, monoaminergic axon loss begins at 12 months of age and increases at 18 months, indicating age- and pathology-dependent degeneration.^42^ Interestingly, the neurodegeneration was not associated with local Aβ deposits, and it was evident with and without tau pathology.^42^ These results suggest that monoaminergic degeneration occurs in the distal Aβ pathology, regardless of tau pathology.^42^ Moreover, hippocampal 5-HT release is disrupted in the 5xFAD mouse model due to Aβ pathology. This has been shown by measuring 5-HT levels in brain slices from 9 to 10 month-old 5xFAD and wild-type (WT) mice following electrical stimulation, in which 5xFAD brain slices showed a 5-HT release of 8.5 nM, compared to WT 5-HT release of 14.6 nM. This reduction is associated with a 14% lower density of 5-HT neurons in 5xFAD mice compared to WT mice. While the 14% decrease in serotonergic neurons is moderate compared to the 42% decrease in 5-HT levels, these findings indicate a reduction in both serotonergic neurons and 5-HT release.^46^ Moreover, the same study highlighted the effects of mitochondrial changes in serotonergic neurons, in which 5xFAD’s 5-HT neurons showed reduced expression of the 5-HT transporter (SERT), mitochondrial fragmentation, and reduced mitochondrial volume. These findings were confirmed in vitro by treating isolated serotonergic neuronal cells with 500 nM oligomeric Aβ.^46^ The in vitro treatment with oligomeric Aβ reduced mitochondrial density and volume, as well as neuritic mitochondrial ATP production, compared with vehicle-treated neuronal cultures.^46^ These results suggest a role for AD in serotonergic degradation and indicate that mitochondrial dysfunction contributes to serotonergic alterations in Aβ pathology.

### Serotonin Receptors and Transporters

Multiple transporters influence 5-HT signaling, including dopamine transporter (DAT), norepinephrine transporter (NET), organic cation transporters (OCT), and the plasma membrane monoamine transporter (PMAT).^47,48^ However, serotonin transporter (SERT) is the primary transporter of 5-HT, which is mainly expressed on the presynaptic membrane of serotonergic neurons.^34,49^ 5-HT has multiple receptors, numbered consecutively from 1 to 7, all of which are G-protein-coupled receptors (GPCRs) except the 5-HT_3_ receptor. The 5-HT_3_ receptor is the only ligand-gated receptor within the family that acts as an ion channel. In this section, we review serotonergic transporters and receptors in health and neuronal degeneration. Figure 2 summarizes serotonin receptors and transporter signaling, along with their main downstream pathways, as described below.

**Figure 2:**
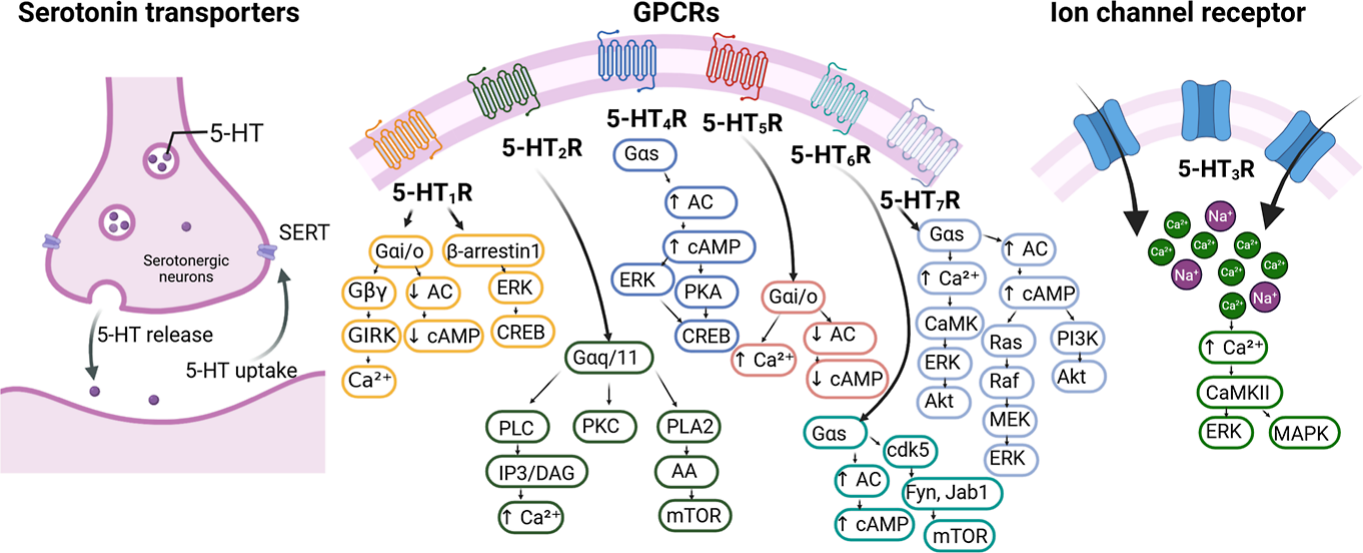
Serotonin signaling pathways mediated by transporters, GPCRs, and ion channel receptors. This diagram summarizes key serotonergic signaling. Left: SERT, the serotonin transporter that regulates synaptic 5-HT levels through reuptake into presynaptic neurons. Center: 5-HT GPCR families and their downstream pathways. Right: The ionotropic 5-HT_3_ receptor functions as a ligand-gated cation channel, causing ion influx and activating downstream signaling. Together, these pathways illustrate the diverse mechanisms by which serotonin modulates neuronal activity and cellular responses.

## Serotonin Transporter (SERT)

SERT, the 5-HT transporter, terminates 5-HT signaling and regulates serotonergic neurotransmission by recycling 5-HT from the synaptic cleft back to the presynaptic neuron.^50^ Blocking SERT inhibits 5-HT uptake, increases extracellular 5-HT, and enhances serotonergic neurotransmission.^34,49^ SERT expression decreases in the aging human brain, as measured by single-photon emission computed tomography (SPECT) in healthy volunteers using the SERT ligand [123I]­2β-carbomethoxy-3β-(4-iodophenyl) tropane ([123I]­β-CIT). The reduction in ligand binding was 29.5% in the aged brain compared to younger subjects, which corresponds to a 4.2% decline in SERT binding per decade.^51^ However, analysis of post-mortem human brain tissue showed mixed results, with some studies indicating no effect or an increase in [3H]­imipramine binding in the frontal cortex, others showing no effect of aging on [3H]­imipramine binding in the temporal cortex, hippocampus, amygdala, and basal ganglia, or an increase in [3H]­imipramine binding in the parietal cortex and hypothalamus, or a reduction in SERT binding in the cingulate cortex.^51–54^ SERT expression in cortical, striatal, and limbic regions is linked to the cognitive abilities of patients with mild cognitive impairment (MCI), with lower SERT levels associated with deficits in auditory-verbal and visual-spatial memory.^55^

In the triple AD transgenic mouse model 3xTg, at ages 3 and 18 months, increased neuron density and more CA1 hippocampal SERT terminals were observed compared to age-matched controls.^56^ This increase in serotonergic sprouting is believed to be a compensatory mechanism, or an intrinsic neuroprotective response to Aβ neurotoxicity.^56^ The reduction in SERT density is associated with neuroinflammation, specifically increased levels of TNFα, IL-1β, and interleukin-6 (IL-6) in 18–24 month-old APPswe/PS 1dE9 mice compared to age-matched controls.^49^ Furthermore, the uptake rate of radioactive 5-HT in 20 month-old neocortex brain sections of APPswe/PS 1dE9 mice is lower compared to WT mice after soluble Aβ40 treatment. These findings suggest that SERT activity may rely on the presence and levels of soluble Aβ peptides, especially the Aβ_40_/Aβ_42_ ratio, which could explain the variability in patients’ responses to antidepressants in AD.^49^

### Serotonin Receptor 1 (5-HT_1_ Receptor)

5-HT_1_ receptors are widely distributed throughout the CNS, including the cortex, hippocampus, basal ganglia, substantia nigra, raphe nuclei, and spinal cord, and are also found in peripheral sensory neurons of the dorsal root ganglia and in components of the brain’s neurovascular unit.^57^ Functionally, 5-HT_1_ receptors act as both autoreceptors on serotonergic neurons and heteroreceptors on nonserotonergic neurons, allowing precise regulation of neuronal excitability and 5-HT release.^58^

All 5-HT_1_ receptor subtypes couple to inhibitory Gαi/o proteins, leading to suppression of adenylyl cyclase activity and reduced intracellular cAMP levels.^59^ Activation of 5-HT_1A_, 5-HT_1B_, and 5-HT_1D_ receptors engages PTX-sensitive Gαi/o signaling, leading to Gβγ-mediated opening of GIRK channels and inhibition of voltage-gated Ca^2+^ channels, thereby hyperpolarizing neurons and decreasing neurotransmitter release.^60,61^ Through these convergent mechanisms, 5-HT_1_ receptors strongly inhibit neuronal firing and synaptic transmission. Additionally, the 5-HT_1E_ receptor uniquely recruits β-arrestin1 through interaction with NFα1/CPE, activating ERK-CREB signaling pathways linked to neuroprotection and cell survival.^60^

The 5-HT_1A_ receptor plays a central role in mood regulation, stress resilience, and neuronal plasticity through interactions with ERK and mTOR signaling pathways.^61^ As a somatodendritic autoreceptor in the dorsal raphe nucleus, the 5-HT_1A_ receptor mediates negative feedback control of serotonergic neuron firing. Acute elevation of extracellular 5-HT, such as after SSRI administration, initially suppresses 5-HT release through autoreceptor activation; with chronic treatment, receptor desensitization occurs, leading to enhanced serotonergic transmission and therapeutic efficacy.^62,63^ In AD, blockade of the 5-HT_1A_ receptor in a streptozotocin (STZ)-induced AD rat model using NAD-299 reduced oxidative stress markers and preserved hippocampal neuronal integrity compared with STZ alone. These findings suggest that inhibiting 5-HT_1A_ receptors may exert neuroprotective effects in AD.^64^ Zimmer et al. examined alterations in hippocampal 5-HT_1A_ receptors expression during the prodromal stages of AD, observing that changes in the serotonergic system occur early and are closely associated with cognitive function.^65^ 5-HT_1A_ receptors are upregulated during the early or prodromal stages of AD, with later reductions as the disease advances. These findings indicate that, in AD, changes in 5-HT_1A_ receptors are dynamic and stage-dependent, complicating therapeutic strategies that aim to either enhance or inhibit 5-HT_1A_ receptors signaling.^65^

Finally, 5-HT_1A_ receptors are also expressed in non-neuronal cells of the neurovascular unit, including endothelial cells and pericytes. In human brain endothelial–pericyte cocultures, activating 5-HT_1A_ receptors increases claudin-5 expression, indicating a role in maintaining BBB integrity.^66^ Disruption of BBB-mediated serotonergic regulation, particularly involving 5-HT_1A_ receptor signaling within the dorsal raphe nucleus, may contribute to serotonergic dysfunction observed in AD. However, the extent to which 5-HT_1_ receptor density or signaling undergoes alterations during the progression of AD remains poorly understood.^67^

### Serotonin Receptor 2 (5-HT_2_ Receptor)

5-HT_2A_ receptors are most highly expressed in the CNS, particularly in the neocortex, entorhinal and piriform cortex, caudate nucleus, nucleus accumbens, olfactory tubercle, and hippocampus.^68,69^ The 5-HT_2_ receptor family includes three receptor types: 5-HT_2A_, 5-HT_2B_, and 5-HT_2C_, all first identified in humans in the early 1990s.^70^ The three subreceptors are GPCRs positively coupled to phospholipase C (PLC), which promotes the production of 1,4,5-trisphosphate (IP3) and diacylglycerol as secondary messengers, or to phospholipase A2 (PLA2), resulting in the release of arachidonic acid (AA) as a secondary messenger.^71,72^

These receptors are best known for their role in the psychoplastogenic mammalian target of rapamycin (mTOR) response to psychedelic drugs and receptor agonists.^73^ Numerous studies have examined the link between AD and the 5-HT_2A_ receptor, showing a connection with decreased receptor density and binding affinity.^74–77^ These studies have explored various aspects of this decline, indicating that serotonergic deterioration is already present in the early stages of the disease. Additionally, comparisons with age-matched controls and healthy young subjects have shown that this decline occurs independently of the onset of AD symptoms.^78^ While reports examining this decline show no correlation with AD symptoms,^78^ a study examining genetic differences in the 5-HT_2A_ receptor gene found a single-nucleotide polymorphism (T102C) that may have functional effects in AD, showing reduced binding affinity compared to age-matched controls with the same polymorphism. This difference is not statistically significant in groups without this genetic variation.^79^

In another study, a mouse carrying a human loss-of-function TPH2 variant that reduces 5-HT synthesis showed impaired cognitive flexibility and increased perseverative behavior.^80^ Treatment with the selective 5-HT_2C_ receptor agonist CP-809,101 partially reversed those deficits, supporting the idea that 5-HT_2C_ receptor activation can mitigate cognitive impairments associated with lower 5-HT levels.^80^ In the brains of AD patients, 5-HT_2_ receptor binding in the cerebral cortex, measured with 18­[18F]­Setoperone PET, is decreased, indicating lower expression levels due to pathology.^74^ However, 5-HT_2C_ receptor expression is higher in natural killer (NK) cells compared to other subtypes.^81^ Martins et al. have shown that increased NK-5-HT_2C_ receptor expression is associated with altered NK cell cytotoxicity, which may contribute to immune system dysregulation.^81^ Moreover, this indicates that NK cell activity could vary in response to serotonergic signaling, making the receptor a potential target for neuroinflammation treatment in AD.

In vitro assessment using mouse 3T3 fibroblast lines overexpressing 5-HT_2A_ and 5-HT_2C_ receptors showed that activation of both receptors stimulates APP ectodomain secretion upon 5-HT exposure. The authors did not distinguish whether the released fragment was sAPPα or sAPPβ, but they interpreted the increase as indicative of enhanced nonamyloidogenic processing. These results suggest that 5-HT_2A_ and 5-HT_2C_ receptors could promote the nonamyloidogenic processing of APP, which might be therapeutically beneficial by reducing Aβ production.^82^ On the other hand, Yuede et al. have demonstrated that treating APP/PS1 mice with pimavanserin, a selective 5-HT_2A_ receptor inverse agonist, decreases Aβ pathology by lowering Aβ production and enhances cognitive function,^83^ an effect that was not observed in 5-HT_2A_ receptor knockout mice.^83^ This discrepancy could be attributed to differences in the models used to study the receptor and the distinct interventions used to modulate its activity.

Finally, a single nucleotide polymorphism (SNP) at position 102T of the 5-HT_2A_ receptor has been correlated with several neuropsychiatric symptoms, including delusions, agitation, and aggression, in comparison to other 5-HT_2_ receptor polymorphisms.^84^ Another study has confirmed a higher frequency of delusions, hallucinations, psychosis, and abnormal motor behaviors in AD patients carrying the C allele or CC genotype of the silent T102C variant of the 5-HT_2A_ receptor. However, the study did not observe the same increase in agitation and aggression as reported in earlier research. Additionally, it has been shown that the C allele and CC genotype frequencies of the cys23ser variant of the 5-HT_2C_ receptor are linked to anxiety and appetite disturbances in female AD patients.^85^

### Serotonin Receptor 3 (5-HT_3_ Receptor)

The 5-HT_3_ receptor is found in both the central and peripheral nervous systems, with the highest concentration in the upper regions of the brainstem and in areas of the brain and body that control the vomiting reflex.^86^ Because the 5-HT_3_ receptor is highly expressed in the brainstem, it has recently emerged as a breakthrough target for treating nausea and vomiting caused by chemotherapy in cancer patients.^87^ The 5-HT_3_ receptor is unique among serotonergic receptors as it is a ligand-gated cation channel rather than a GPCR. It is a cys loop receptor, containing five identical or nonidentical molecular subunits that surround a water-filled ion channel in the center of the structure.^88^

These subunits are labeled alphabetically, 5-HT_3A_, 5-HT_3B_, 5-HT_3C_, 5-HT_3D_, and 5-HT_3E_.^88^ Each of these units is distinct from the other, and none can function or exhibit activity without the presence of at least one 5-HT_3A_ receptor subunit.^89^ The 5-HT_3A_ receptor mediates rapid activation and desensitization by initiating an inward ion current (ion influx).^88^ 5-HT_3B_ receptors do not form functional homomeric receptors but can coexpress with A subunits to produce heteromeric 5-HT_3AB_ receptors.^89^ Upon activation of the 5-HT_3_ receptor, the channel opens, allowing rapid influx of Na^+^ and Ca^2+^, producing fast excitatory postsynaptic responses.^90^ Moreover, at the molecular level, 5-HT_3_ receptor activation increases intracellular Ca^2+^ by increasing Ca^2+^ influx and mobilizing Ca^2+^ from intracellular stores.^91^ Ca^2+^ influx activates calmodulin-dependent protein kinase II (CaMKII)/ERK and triggers mitogen-activated protein kinase (MAPK) cascades to modulate inflammatory response.^92–94^

The 5-HT_3_ receptors have promising neuroprotective effects when targeted with tropisetron, a 5-HT_3_ receptor antagonist with partial agonist activity at the α7-nicotinic acetylcholine receptor (α7 nAChR).^95^ Under the influence of the antagonist, many common risk factors for AD development were mitigated or reduced. These include inhibition of pro-inflammatory nuclear factor kappa-light-chain-enhancer of activated B cells (NF-κB), increased leukocyte transmigration into the brain, and reduced tumor necrosis factor alpha (TNFα) level.^95^ In AD, Barnes et al. showed no change in 5-HT_3_ receptor recognition site density in the amygdala and hippocampus of AD patients, suggesting that 5-HT_3_ receptor expression is stable under pathology.^96^ In this study, the 5-HT_3_ receptor density was measured using [^3^H]-(S)-zacopride binding, with nonspecific binding defined by competition with granisetron.^96^ Marazziti et al. showed that the 5-HT_3_ receptor antagonist ondansetron has neuroprotective and anti-inflammatory effects in vitro, where immortalized ApoE3 and ApoE4 astrocytes exhibited increased ApoE secretion following ondansetron treatment, especially in ApoE3 cells, through activation of the Liver X Receptor–ATP-binding cassette transporter A1 (LXR-ABCA1) pathway.^97^ These findings indicate that 5-HT_3_ receptor activity may be altered in AD pathology despite stable expression, and a similar increase in ApoE secretion was also observed in primary human ApoE3/3 and ApoE4/4 astrocytes.^97^ However, although ondansetron demonstrated neuroprotective effects through multiple mechanisms, it did not improve cognitive function in AD patients at doses of 20 μg/day or 100 μg/day after 24 weeks of treatment.^98^ Tropisetron showed similar in vitro neuroprotective effects.^99^ Tropisetron treatment increases sAPPα and decreases Aβ_42_ in primary hippocampal neuronal cultures at 1 μM. This increase was observed in the presence of both ApoE3 and ApoE4 transfection.^99^ In J20 mice, subcutaneous injection of 0.5 mg/kg/day tropisetron for 2 months improved spatial memory as measured by the Morris water maze and increased brain sAPPα levels. Finally, comparing the effects of tropisetron to memantine, both enhanced cognitive abilities, with tropisetron being superior, which could be attributed to its ability to bind multiple receptors, namely the α7 nAChR and 5-HT_3_ receptor.^99^ The mechanisms by which tropisetron exerts its beneficial effects may be attributed to its ability to modulate neuronal firing in pyramidal and fast-spiking hippocampal CA1 cells, thereby restoring neuronal network balance.^100^ However, the authors did not evaluate whether the effect is mediated by the 5-HT_3_ receptor, the α7 nAChR, or both.^100^ Moreover, tropisetron can inhibit calcineurin phosphatase activity and exert antiapoptotic and anti-inflammatory effects, as evidenced by reductions in cyclooxygenase-2 (COX-2), active cysteine-aspartic protease 3 (caspase-3), and inducible nitric oxide synthase (iNOS) expressions in the hippocampus of rats injected with Aβ_42_ peptide.^101^ Although 5-HT_3_ receptor antagonists show positive effects in preclinical studies, more research is needed to translate these findings into clinical practice in AD patients. Granisetron, another selective 5-HT_3_ receptor antagonist, showed similar neuroprotective, antioxidant, and anti-inflammatory effects in both the radiation-induced brain injury rat model and the AD mouse model.^102^ Recently, we demonstrated that granisetron improves the functionality of an in vitro BBB-endothelium model, as measured by the lucifer yellow permeability assay.^103^ This enhancement in BBB tightness persisted even after Aβ treatment. Additionally, we showed that the improved BBB function is partly due to increased expression of tight junction proteins.^103^ Moreover, we have shown that one month of treatment with granisetron (3 mg/kg/day) in TgSwDI mice enhanced BBB function and reduced AD pathology.^104^ Granisetron-treated mice showed a reduction in brain Aβ load by shifting Aβ processing toward the nonamyloidogenic pathway. Additionally, it decreased glial cell activation, increased synaptic markers, and improved cognitive functions in the mice.^104^ These changes in pathology were linked to modulation of the CaMKII/CREB pathway.^103,104^ These findings indicate that targeting 5-HT_3_ receptor could be a potential treatment for AD.

### Serotonin Receptor 4 (5-HT_4_ Receptor)

The 5-HT_4_ receptors are expressed in the GI tract, bladder, ileum, esophagus, heart, and colon, as well as in the brain, primarily in the basal ganglia, cortex, and hippocampus. As such, it has been identified as an important pharmacological target for GI tract motility, bladder function, and heart rate.^105,106^ The 5-HT_4_ receptor is a GPCR that activates adenylyl cyclase, increasing intracellular cAMP levels and triggering protein kinase A (PKA)-dependent phosphorylation cascades and CREB pathways that support neurogenesis and neuroprotection.^107^ In addition, 5-HT_4_ receptor stimulation activates the ERK1/2 pathway in neurons via an independent PKA-related mechanism^108^

In the CNS, the 5-HT_4_ receptor has been associated with functions such as learning, anxiety, and memory.^109^ Within the brain, it is mainly concentrated in the basal ganglia, the hippocampal formation, and the cortical mantle.^109^ Highly specific medications targeting 5-HT_4_ receptors have been synthesized that can freely cross the BBB. However, a definitive full brain agonist or antagonist has yet to be developed.^110^ 5-HT_4_ receptor has established itself as a potential candidate for future treatments of AD, as it is linked to APP processing and Aβ generation in rodent models of AD.^111^ In vitro culture of Chinese hamster ovary (CHO) cells stably expressing the human 5-HT_4E_ receptor isoform showed increased 5-HT_4E_ receptor expression and sAPPα secretion in cell culture media compared to WT cells.^112^ Furthermore, activating the receptor with 5-HT treatment increased levels of nonamyloidogenic sAPPα, as shown by higher sAPPα secretion into the cells’ media. Additionally, treating transfected cells with the 5-HT_4E_ receptor antagonist GR113808 reduced sAPPα secretion.^112^ These results were replicated in IMR32 human neuroblastoma cells, where secreted sAPPα increased as a result of treatment with selective agonists such as prucalopride and Renzapride,^112^ suggesting a role for the 5-HT_4_ receptor in regulating sAPPα secretion, where its activation shifts APP processing toward the nonamyloidogenic pathway, which could have therapeutic value in AD. Moreover, 5-HT_4_ receptor activation alleviates some of the AD-related cognitive deficits.^113,114^ In 5xFAD, an AD mouse model, stimulation of the 5-HT_4_ receptor increased acetylcholine levels in the brain, leading to pro-cognitive effects as determined by enhanced learning and memory.^113,114^ This study highlights the 5-HT_4_ receptor as a pharmacological tool and demonstrates that 5-HT_4_ receptor agonists enhance performance on memory tasks in rodents, while receptor antagonists impair the performance.^113,114^ Long-term administration of the 5-HT_4_ receptor agonist RS67333 reduced Aβ pathology by increasing the nonamyloidogenic cleavage of APP, thereby promoting the neurotrophic protein sAPPα and alleviating AD pathology and reducing Aβ plaques.^113,114^ Furthermore, early administration of the selective agonist RS67333 during asymptomatic AD in 5xFAD mice reduced brain Aβ load, hippocampal astrogliosis and microgliosis, temporarily increased brain and CSF sAPPα levels, and improved cognitive function in the mice, suggesting that 5-HT_4_ receptor agonists may have potential therapeutic effects for AD.^113,114^ Comparable therapeutic effects have been observed in treating the PS19 mouse model of tauopathy with 5-HT_4_ receptor agonists; mice treated with prucalopride (a partial agonist at 3 mg/kg) or RS67333 (a selective agonist at 2 mg/kg) showed reductions in tau pathology, improvements in proteasome proteolytic capacity, and alleviation of specific cognitive deficits, particularly anxiety-like behaviors and hippocampal-dependent spatial memory.^115^

### Serotonin Receptor 5 (5-HT5 Receptor)

The 5-HT_5_ receptor family, first discovered in 1992, includes the 5-HT_5A_ and 5-HT_5B_ subreceptors; however, only 5-HT_5A_ receptor is expressed in humans, as the gene encoding the 5-HT_5B_ receptor is interrupted by stop codons.^116^ The 5-HT_5A_ receptor is primarily found in the cerebral cortex, amygdala, hippocampus, and cerebellum.^117^ Activation of the 5-HT_5_ GPCR causes inhibition of adenylyl cyclase and an increase in intracellular Ca^2+^ levels.^118^

Numerous studies have explored the link between 5-HT_5A_ receptor gene variation and susceptibility to schizophrenia and other conditions, such as depression and bipolar disorder.^119–121^ However, results have been inconsistent, with some studies finding no association while others report a significant correlation.

A previous functional investigation showed that the proposed antinociceptive effect of serotonergic receptors could be triggered by nonselective agonists, and reduced by 5-HT_5A_ receptor-specific antagonists such as SB-699551 A.^122^ This finding consequently suggests that activating the 5-HT_5A_ receptor produces antinociceptive effects. A similar study using the same antagonist, SB-699551 A, and the nonselective agonist l-tryptophan found that inhibition of the 5-HT_5A_ receptor led to decreased short- and long-term memory performances, whereas l-tryptophan improved performance and attenuated the effect of the selective antagonist SB-699551 A.^123^ However, a study employing three novel 5-HT_5A_ receptor antagonists, namely ASP5736, AS2030680, and AS2674723 with high affinity for the 5-HT_5A_ receptor, demonstrated that all compounds inhibited drug-induced memory impairment.^124^ These findings imply that the receptor antagonism may enhance working memory and could be applicable in the treatment of cognitive impairment and dementia.^124^

### Serotonin Receptor 6 (5-HT_6_ Receptor)

The 5-HT_6_ receptor is a GPCR primarily found in the CNS. Its expression in the CNS and absence in peripheral tissues have led to its identification as a promising novel target for therapeutic intervention in cognitive function.^125^ In vitro and in vivo studies have shown that blockade of 5-HT_6_ receptors is associated with enhanced release of acetylcholine and glutamate, as well as changes in cAMP levels.^126,127^ Moreover, 5-HT_6_ receptor expression regulates Cyclin-dependent kinase 5 (Cdk5).^128^ Through regulation of Cdk5, the 5-HT_6_ receptor also influences the expression of the Fyn proto-oncogene (Fyn), a member of the Src family tyrosine kinases, which controls T cell and B cell receptor signaling; Jun activation domain-binding protein 1 (Jab1), which promotes cell growth; and mTOR function.^129^ Specifically, blocking the 5-HT_6_ receptor in rodents has been shown to decrease mTOR overactivation, a signaling pathway that regulates cell proliferation and cell death.^130^ 5-HT_6_ receptor antagonists can produce promnesic and/or antimnesic effects.^131^ This includes memory formation, cognitive impairments due to age, and memory deficits in models for diseases such as schizophrenia, Parkinson’s disease, and AD. Nevertheless, although targeted antagonism of this receptor has demonstrated a great potential for therapeutic benefit, it failed to confirm robust cognitive benefits in clinical trials.^131^ In AD, changes in 5-HT_6_ receptor expression have been reported. Yet, the direction and clinical significance of this association remain unclear, as both agonists and antagonists have been shown to enhance rodent cognitive function.^130^ PRX-07034, a selective 5-HT_6_ receptor antagonist, administered at 1 and 3 mg/kg in rats, improved learning, as measured by strategy switching and spontaneous alternation performance in a cross-maze.^132^ Developed by Avineuro Pharmaceuticals, AVN-322 is a selective 5-HT_6_ receptor antagonist intended for treating AD and schizophrenia. It has demonstrated effectiveness in enhancing cognitive function and can be administered with minimal or no adverse effects.^131^ These, along with other recently discovered and researched 5-HT_6_ receptor antagonists such as SAM-760, Idalopirdine, and others, constitute a novel class of medications to be introduced.^131^ Because of their procholinergic properties, these medications are often used as adjuncts to AchEIs, further increasing acetylcholine levels in the CNS.^133,134^ Positive effects were observed in early clinical trials, where some 5-HT_6_ antagonists, such as idalopirdine and SB 742457, demonstrated improvements in cognition and activities of daily living; however, larger Phase III trials failed to show significant clinical benefits, indicating that further development is needed, possibly including combination therapy or alternative dosing strategies.^131^ Treatment with the 5-HT6 receptor agonist, E-6801 (2.5 mg/kg), improved male Lister hooded rats’ behavior in the conditioned emotional response paradigm in scopolamine-induced cognitive impairment. While E-6801 alone did not significantly alter conditioned freezing behavior, it significantly reversed scopolamine-induced deficits in contextual memory, showing that 5-HT_6_ receptor activation enhances associative learning.^135^

### Serotonin Receptor 7 (5-HT_7_ Receptor)

The most recently identified subtype is the 5-HT_7_ receptor, a GPCR positively linked to adenylate cyclase activation, which was identified in 1993.^136^ The receptor has four different isoforms from A to D.^137^ The only functional difference between the isoforms is that the 5-HT_7D_ receptor exhibits a different pattern of internalization compared to 5-HT_7A_ and 5-HT_7C_ receptors.^138^ The activation of the 5-HT_7_ receptor increases cAMP production and intracellular Ca^2+^ levels.^139^ The increase in cAMP leads to the Ras-Raf-MEK-ERK signaling cascade and the phosphoinositide-3-kinase (PI3K)/Akt pathway.^137,139^ Whereas the intracellular Ca^2+^ increase activates the Ca^2+^/CaMK pathway, causing ERK and Akt phosphorylation.^140^ These signaling pathways enhance the expression and phosphorylation of the tropomyosin receptor kinase B (TrkB) receptor, which promotes neuronal survival, synaptic plasticity, and neurotrophic support, suggesting a neuroprotective effect of 5-HT_7_ receptor activation.^137,139,140^

One of the most observed functions of the 5-HT_7_ receptor is the regulation of the circadian rhythm, which was previously believed to be mediated by the 5-HT_1A_ receptor.^141^ This is supported by the receptor’s localization in the suprachiasmatic nucleus, an area responsible for mammalian circadian rhythms.^142^ In the peripheral system, the 5-HT_7_ receptor has been proposed to inhibit GI peristalsis.^143^ Despite high interspecies homology (95%), the 5-HT_7_ receptor shows low sequence homology with other serotonergic receptor subtypes (<40%).^144^

The 5-HT_7_ receptor has also been investigated in schizophrenia owing to its localization within thalamic and limbic structures, as well as its high binding affinity to several established antipsychotic and antidepressant medications.^145^ Nonetheless, only a limited number of studies have investigated the association between the 5-HT_7_ receptor and AD. One such investigation found that 5-HT_7_ receptor mRNA levels in the Brodmann area 10 (BA10) region of the brain, which is responsible for decision-making and working memory, decreased 5-fold in AD, while showing a consistent increase in the thalamus. However, no significant correlation was found between 5-HT_7_ receptor mRNA levels and dementia stage or cognitive assessments.^146^ Deletion of the 5-HT_7_ receptor gene in wild-type mice resulted in memory impairment, which was more pronounced in aged (24 months) than in young (3 months) mice.^147^ Furthermore, the deletion of 5-HT_7_ receptors led to an increase in 5-HT_1A_ and 5-HT_2A_ receptors in the hippocampus, suggesting a compensatory response mechanism.^147^

### Serotonin Influence on the BBB

Previous research has demonstrated that 5-HT functions as a vascular modulator.^148^ 5-HT exerts vasoconstrictive effects by directly activating smooth muscle cells, stimulating norepinephrine release, and enhancing the action of other endogenous vasoconstrictors, such as catecholamines.^148,149^ Furthermore, 5-HT may also induce vasodilation by activating endothelial cells, inhibiting adrenergic signaling pathways, reducing smooth muscle cells contraction, or stimulating the release of vasodilators.^148,149^ The influence of 5-HT on vascular contractility is affected by various local and chronic factors, including temperature, injury, blood pressure, and the structural and functional integrity of the vessel’s cellular components, especially the endothelium and smooth muscle cells.^148,149^ Moreover, in the periphery, upon injury or platelet activation, 5-HT is released from platelets to promote aggregation along with vasoconstriction or dilation.^150^

Within the CNS, selective serotonin reuptake inhibitors (SSRIs) have been reported to affect the endothelial function. In patients diagnosed with depression, treatment with escitalopram for 8 or 24 weeks decreased levels of circulating endothelial cells, as well as soluble vascular cell adhesion molecule-1 (VCAM-1) and von Willebrand factor (VWF) in blood samples, indicating less endothelial cells damage. This reduction is associated with decreased inflammation and oxidative stress, along with the restoration of endothelial nitric oxide synthase (eNOS), showing the ability of SSRIs to restore the vasoconstrictive function of in vitro cultured endothelial cells exposed to patient sera.^151^ A meta-analysis of patients with depression undergoing treatment with SSRIs determined that SSRI therapy enhanced endothelial cells function, as measured by flow-mediated dilation (FMD).^152^ FMD is recognized as the gold standard technique for evaluating arterial endothelial function.^152,153^

BBB dysfunction is increasingly acknowledged as a key and early aspect of AD development, rather than just a secondary result of neurodegeneration.^154,155^ Neuroimaging and neuropathological investigations have established that the breakdown of the BBB frequently initiates in the hippocampus and other susceptible regions during AD. This phenomenon correlates with cognitive decline and occurs independently of substantial Aβ or tau pathology.^156–158^ At the molecular level, BBB dysfunction in AD involves the breakdown of the cerebrovascular unit: loss of endothelial tight junction proteins, pericyte degeneration, and degradation of the capillary basement membrane, all of which contribute to increased barrier permeability.^159,160^ Consistent with these effects, AD brains show perivascular deposits of blood proteins and capillary degeneration indicative of chronic BBB leakage.^157,159^ Notably, individuals carrying the ApoE4 experience accelerated pericyte loss, microvascular injury, and BBB breakdown relative to noncarriers.^156,158^ 5-HT system modulators have been shown to modify the BBB; pretreatment with *p*-chlorophenylalanine (*p*-CPA), a 5-HT synthesis inhibitor, has been shown to improve the BBB functionality, reduce brain edema, and restore cerebral blood flow in a rat model of brain trauma.^161^ In rats, 5-HT contributes to BBB breakdown via the 5-HT_2_ receptor.^162^ Rats subjected to short-term forced swimming exercise exhibited elevated levels of 5-HT in both the brain and plasma. These stress-induced increases are correlated with BBB dysfunction, as assessed by Evans blue albumin and 131I-sodium permeation.^162^ Moreover, reductions in 5-HT levels, achieved either through treatment with *p*-CPA or through the destruction of serotonergic neurons with 5,7-dihydroxytryptamine (5,7-DHT), resulted in a reduction in BBB impairment,^162^ indicating that excessive serotonergic signaling, rather than 5-HT itself under physiological conditions, drives BBB disruption.

Finally, although it has been reported that 5-HT does not cross the BBB, a recent study showed that 5-HT is transported into the brain via SERT expressed on the endothelial cells.^163^ Furthermore, when 5-HT is internalized, it can diffuse through the tight junctions of endothelial cells, forming a coupled endothelial syncytium and creating a short-circuit path within the barrier layer. These findings are significant because this coupling can enhance vasodilation throughout the vasculature, enabling signals to travel from capillaries to arteries, underscoring the role of 5-HT in regulating brain vasculature and blood flow.^163^

### Serotonin and Gut-Brain Axis

The gut-brain axis is a two-way communication system where the brain and gut influence and interact with each other. This system is essential for maintaining GI balance and also plays a key role in connecting aspects of brain function, such as emotions and higher cognitive processes.^164^ It is connected anatomically through the CNS, the autonomous nervous system (ANS), the hypothalamic-pituitary adrenal (HPA) axis, the enteric nervous system (ENS), and by the innervation of the GI tract.^164,165^

There are numerous mechanisms by which gut bacteria influence the brain, including the HPA axis, the vagus nerve, the secretion of short-chain fatty acids (SCFAs), the modulation of BBB permeability, and the production and regulation of neurotransmitters.^166^ Bacteria have been demonstrated to produce a wide array of neurotransmitters, including dopamine, noradrenaline, acetylcholine, GABA, and 5-HT.^166^ Figure 3 demonstrates an overview of the gut-brain axis of 5-HT in health and in disease. Moreover, gut microbiota can produce metabolites that modify the serotonergic system.^104,167,168^ Researchers have shown that granisetron, a 5-HT_3_ receptor antagonist, is produced by the gut microbiota, mostly by *Clostridium difficile* and *Citrobacter freundii*, and to a lesser extent, by *Bacteroides thetaiotaomicron* and *Paenibacillus polymyxa*, which are known to be altered in AD.^169–173^ Interestingly, the production of a 5-HT_3_ receptor antagonist by the gut microbiota is induced by external granisetron treatment, without affecting the bacterial diversity.^171^

**Figure 3:**
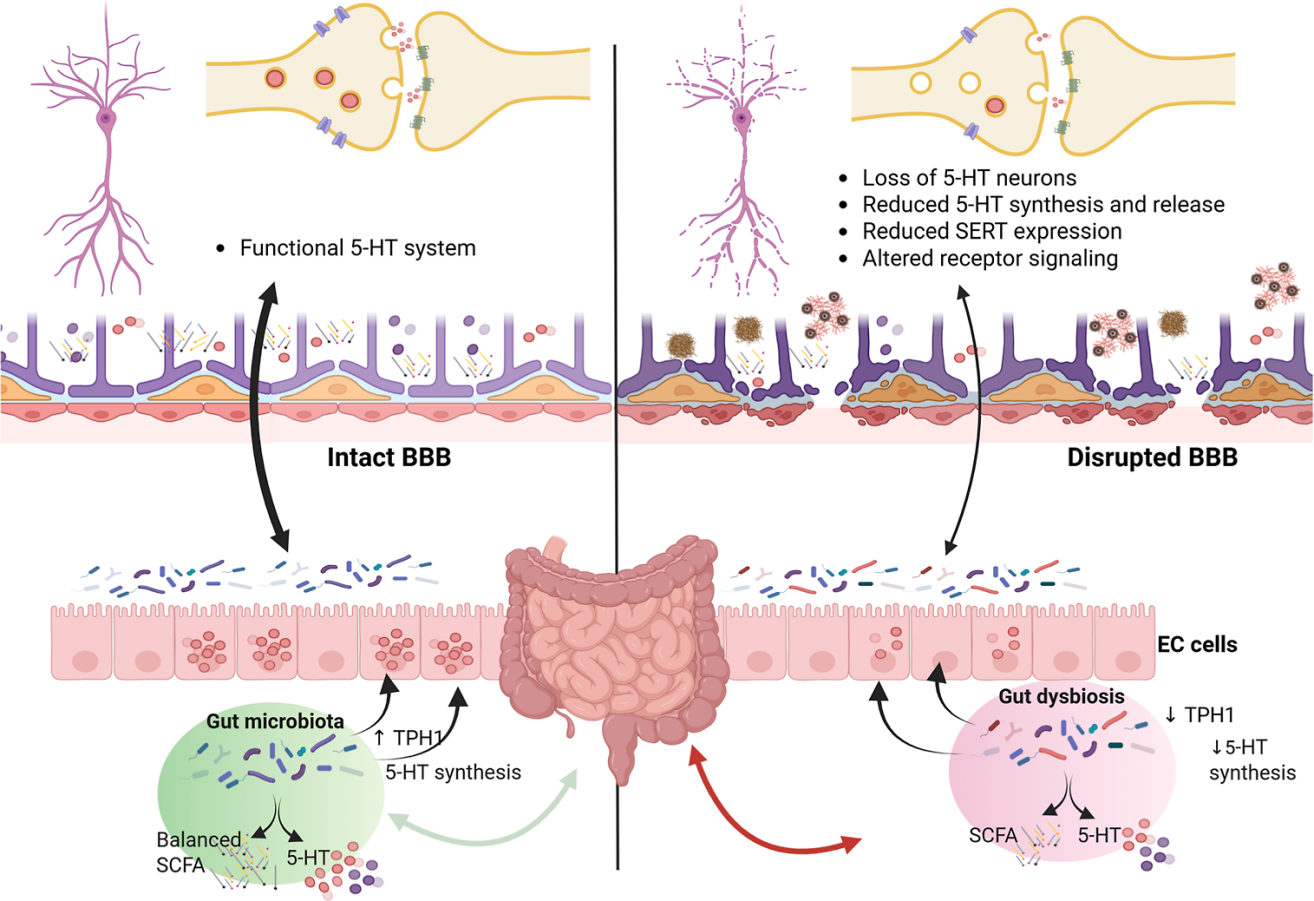
Gut–brain serotonergic signaling in healthy conditions and AD pathology. The left panel shows a healthy state where diverse gut microbiota promotes short-chain fatty acid (SCFA) production, generates serotonin metabolites, activates TPH1 in enterochromaffin (EC) cells, and supports normal peripheral 5-HT synthesis. Gut serotonin influences the brain through strong vagal nerve signaling, the HPA axis, and the passage of SCFA and, to a lesser extent, 5-HT across an intact BBB, enabling proper central serotonergic neuron function. The right panel illustrates AD-related pathological conditions, including neuronal death, BBB damage, gut dysbiosis, and serotonin dysregulation. In AD, gut dysbiosis alters SCFA levels, reduces TPH1 activity, and decreases 5-HT synthesis in enterochromaffin cells. This changed peripheral environment, combined with a disrupted BBB, contributes to serotonergic deficits, including loss of 5-HT neurons, decreased neuronal 5-HT synthesis and release, lower SERT expression and activity, and impaired receptor signaling. Arrows indicate bidirectional communication linking gut microbial signals, peripheral 5-HT, and serotonergic balance.

About 90 to 95% of 5-HT is synthesized and stored in the GI tract.^166^ This localization of 5-HT suggests that the gut microbiome modulates 5-HT. Studies have reported reduced blood and colon levels of 5-HT in germ-free mice in comparison to controls.^174^ Many bacteria can produce 5-HT,^166^ either directly by producing 5-HT, or indirectly by increasing TPH1 expression, or producing tryptophan, tryptamine, and similar monoamines.^175,176^ The impact of the gut microbiome appears to be related to the production of SCFA, specifically butyrate and acetate,^177^ which signals the enterochromaffin cells to produce 5-HT via the expression of TPH1.^175,178^ Butyrate is a much more potent SCFA for this pathway, with 0.5 and 1 mM sodium butyrate treatment resulting in a 3.5- and 2.5-fold increase in TPH1 production, respectively, whereas treatment of 10–50 mM sodium acetate had a greater than 2-fold increase, with a maximal increase of 3.2-fold at 30 mM.^177^ Propionate is another prominent SCFA that contributes to 5-HT upregulation. In a study by Nankova et al., the treatment of rat pheochromocytoma of sympathoadrenal origin (PC12 cells) with 1 mM propionate and butyrate significantly increased tyrosine hydroxylase (TH) protein levels, the rate-limiting enzyme in catecholamine synthesis, with butyrate having the more potent effects.^179^ Moreover, propionate specifically induced the 5-HT-synthesizing enzyme TPH1 and cofactor guanosine triphosphate cyclohydrolase (GTPCH), linking it directly to enhanced 5-HT production.^179^ While butyrate is more potent for this mechanism, it is worth mentioning that acetate is the most abundant, where acetate, propionate, and butyrate typically appear in an approximate molar ratio of 60:20:20, respectively.^180^ The potency of butyrate in this pathway, combined with the implications of compositional differences in butyrate-producing bacteria under conditions related to 5-HT, makes butyrate a primary target for treating conditions associated with 5-HT dysregulation, such as mood disorders and cognitive impairments.

The importance of the gut’s impact on 5-HT levels is highlighted in research related to anxiety and depression; both conditions are associated with microbial imbalances and dysregulation of 5-HT within the GI system.^165^ Although anxiety and depression are both intricate conditions, their relationship is extensively documented, and their connection to 5-HT and dysbiosis, and SCFAs has garnered increasing recognition. In C57BL/6J male mice subjected to chronic psychosocial stress, the administration of SCFAs, namely, propionate, acetate, and butyrate, mitigated behaviors resembling depression and anxiety and improved the mice’s stress responsiveness.^181^ Another study, conducted by Valles-Colomer et al. (2019), examined correlations in gut composition with quality-of-life and depression metrics. The researchers found a positive link between butyrate-producing *Faecalibacterium* and *Coprococcus*, and an overall higher quality of life, as well as a depletion of *Dialister* and *Coprococcus* species in cases of depression, specifically.^182^ In another study comparing fecal bacterial composition between major depressive disorder (MDD) and healthy controls, *Firmicutes* was reduced in MDD.^183^ In the case of anorexia nervosa (AN), Zhao et al. (2024) conducted a systematic review of studies on patients with AN and found significantly different gut microbiome compositions compared to healthy controls. Among the reported studies, four studies assessed the Beck Depression Inventory (a 21-item self-report questionnaire used to measure depression severity) and showed that AN patients experienced at least mild depression. Importantly, among these studies, patients with anxiety or depression exhibited a negative correlation between fecal butyrate levels and anxiety/depression scores.^184^ These findings not only show that anorexia, depression, and anxiety are linked to microbial dysbiosis but also highlight the importance of the microflora in producing SCFAs, which are responsible for most of the body’s 5-HT. These conditions are part of the growing list of issues related to disturbances in the gut microbiome and 5-HT regulation.

In AD, 5-HT-producing microbes are altered. Vogt et al. conducted a comparative study on the gut microbiome of individuals with AD and healthy controls, matched for age, sex, ethnicity, Body Mass Index (BMI), and diabetes status.^173^ Their findings indicated significant reductions in microbial richness (the total number of unique species within a participant) and alpha diversity (the diversity within an individual, considering richness and abundance) among AD patients. Furthermore, they observed notable differences in beta diversity (the similarity or dissimilarity in microbial composition across participants), characterized by a decreased abundance of *Firmicutes* and *Actinobacteria*, and an increased abundance of *Bacteroidetes* in AD patients.^173^ In the phylum *Firmicutes*, the genus *Clostridium* was reduced, which is the dominant producer of butyrate in the colon.^173,185^ Given the association between gut dysbiosis and 5-HT production in patients with AD, the microbiome has emerged as a potential therapeutic target for neurotransmitter dysregulation. Further research is warranted to elucidate the specific role of altered microbiota in the serotonergic system in AD.

Finally, the gut-brain axis is a bidirectional pathway between the brain and gut. The brain can influence the GI through multiple mechanisms.^186,187^ 5-HT produced in the gut can stimulate the afferent vagus nerve fibers, causing DRN serotonergic neuronal modulation as well as the norepinephrinergic neurons in the locus coeruleus.^187^ This is evident as SSRI oral treatment in BALB/c mice postsubdiaphragmatic vagotomy increased afferent vagal fiber activity.^188^ SSRIs (sertraline or fluoxetine) significantly increased the firing frequency of vagal afferent fibers; an effect not observed in mice treated with bupropion, a noradrenaline-dopamine reuptake inhibitor.^188^ Moreover, when comparing the efficacy of SSRI treatment in mice with or without vagotomy, SSRI did not reduce immobility in the tail suspension test, suggesting that the effect of SSRIs, at least in part, depends on vagus-nerve-dependent gut-brain signaling.^188^ This is important, as vagal nerve stimulation in 14 AD patients improved their attention and speech within three months.^189^

## Conclusion and Final Remarks

AD is characterized not only by Aβ and tau pathology but also by significant disturbances in neurotransmitters and disruption of neurovascular integrity. These disturbances include changes in the serotonergic system, ranging from alterations in neurotransmitter levels and metabolism to receptor function and expression, 5-HT transport, and ultimately serotonergic neuronal death. In this review, we provided an overview of the pathology and explored serotonergic changes. Importantly, we highlighted 5-HT’s roles beyond neurotransmission, emphasizing its dual effects as a vasodilator and vasoconstrictor. We also discussed examples of 5-HT affecting BBB integrity under various pathological conditions, such as AD, stress, and brain trauma. Additionally, we briefly reviewed the role of microbiota in modulating the serotonergic system. The gut–brain axis is increasingly recognized as a key regulator of serotonergic signaling and AD pathology. Since 5-HT is mainly produced in the GI tract, gut microbiota can influence its availability, metabolism, and receptor sensitivity both peripherally and centrally. Changes in the microbial ecosystem have been linked to inflammation and shifts in neurotransmitters. These changes may result from an increase in pathogenic bacteria that require antibiotics or a decrease in beneficial bacteria, both of which could be addressed with probiotic supplements. Although our primary focus was on the direct impact of the microbiota on the serotonergic system, it may also indirectly influence this system by modulating inflammation, a topic not covered in this review.

Future research on the serotonergic system should include studies of neurotransmitter dynamics, particularly signaling pathways among receptor subtypes in the AD brain. A better understanding of how 5-HT, its receptors, and transporters change across different brain regions and cellular components as the disease progresses is essential for understanding their role in the disease and for developing more targeted treatments. Additionally, the role of 5-HT in BBB function and in gut-brain axis signaling warrants further investigation. Future studies could reveal how peripheral 5-HT influences CNS levels via BBB transport or by modulating 5-HT synthesis and metabolism. This knowledge is key to gaining a comprehensive understanding of AD pathophysiology. These integrated insights could lead us to new therapeutic targets that restore the brain’s neurochemical balance, preserve vascular integrity, and maintain cognitive function, thereby protecting against AD.
